# Cardio-protective effects of carnitine in streptozotocin-induced diabetic rats

**DOI:** 10.1186/1475-2840-5-2

**Published:** 2006-01-19

**Authors:** John I Malone, David D Cuthbertson, Michael A Malone, Douglas D Schocken

**Affiliations:** 1 The Department of Pediatrics, University of South Florida, College of Medicine, Tampa, FL 33612, USA; 2 The Department of Pediatrics, University of South Florida, College of Medicine, Tampa, FL 33612, USA; 3 The Department of Internal Medicine, University of South Florida, College of Medicine, Tampa, FL 33612, USA; 4 The Department of Internal Medicine, University of South Florida, College of Medicine, Tampa, FL 33612, USA

## Abstract

**Background:**

Streptozotocin-induced diabetes (STZ-D) in rats has been associated with carnitine deficiency, bradycardia and left ventricular enlargement.

**Aim:**

The purpose of this study was to determine whether oral carnitine supplementation would normalize carnitine levels and cardiac function in STZ-D rats.

**Methods:**

Wistar rats (48) were made hyperglycemic by STZ at 26 weeks of age. Same age normal Wistar rats (24) were used for comparison. Echocardiograms were performed at baseline 2, 6, 10, and 18 weeks after STZ administration in all animals. HbA1c, serum carnitine and free fatty acids (FFA) were measured at the same times. Since STZ-D rats become carnitine deficient, 15 STZ-D rats received supplemental oral carnitine for 16 weeks.

**Results:**

The heart rates for the STZ-D rats (290 ± 19 bpm) were less than control rats (324 ± 20 bpm) (p < 0.05). After 4 weeks of oral carnitine supplementation, the serum carnitine and heart rates of the STZ-D rats returned to normal. Dobutamine stress increased the heart rates of all study animals, but the increase in STZ-D rats (141 ± 8 bpm) was greater than controls (79 ± 8 bpm) (p < 0.05). The heart rates of STZ-D rats given oral carnitine, however, were no different than controls (94 ± 9 bpm). The left ventricular mass/body weight ratio (LVM/BW) in the diabetic animals (2.7 ± 0.5) was greater than control animals (2.2 ± 0.3) (p < 0.05) after 18 weeks of diabetes. In contrast, the LVM/BW (2.3 ± .2) of the STZ-D animals receiving supplemental carnitine was the same as the control animals at 18 weeks.

**Conclusion:**

Thus, supplemental oral carnitine in STZ-D rats normalized serum carnitine, heart rate regulation and left ventricular size. These findings suggest a metabolic mechanism for the cardiac dysfunction noted in this diabetic animal model.

## Background

Cardiovascular disease is the most common serious complication of diabetes mellitus. Coronary atherosclerosis and cardiomyopathy occur as a result of the metabolic abnormalities associated with diabetes [1]. These physical changes require years to develop in humans following the onset of chronic hyperglycemia [2]. We have previously shown that cardiac function is altered after 6 months of hyperglycemia in Wistar rats [3]. We and others [4,5] have noted bradycardia to be a consistent feature when evaluating diabetic rat models. Heart rate variability in diabetes is commonly attributed to associated neuropathy [6]. It has been noted, however, that the induction of hyperglycemia after 6 months of life in Wistar rats does not alter peripheral nerve function for an additional 6 months [7]. Carnitine deficiency is often encountered in diabetes mellitus [7,8]. Structural changes in myocardial mitochondria have been associated with diabetes and concomitant carnitine deficiency [3]. In the current study, the evaluation of cardiac functional changes in Wistar rats began with the induction of hyperglycemia at 6 months of age and was monitored on a monthly basis for an additional 18 weeks. The first objective of this study was to observe the evolution of cardiac dysfunction in streptozotocin diabetic (STZ-D) rats to help understand its pathogenesis. Secondly, the effect of carnitine supplementation upon the cardiac dysfunction of diabetes in these animals was evaluated.

## Methods

Male Wistar rats at 26 weeks of age were entered into this study. There was one animal per cage, and each had free access to food and water. The water bottles were filled twice each day and the animals were weighed once each week to insure weight gain and appropriate hydration. Hyperglycemia was induced in 48 animals at 26 weeks of age by the intraperitoneal injection of STZ (35 mg/kg) buffered in cold sodium citrate (pH 4.5). The current investigation conformed to the Guide for the Care and Use of Laboratory Animals [9], and was approved by the Institutional Animal Care and Use Committee at the University of South Florida. Seven days after STZ injection, hyperglycemia was documented by measuring the glucose content of tail vein blood with an Ames Glucometer 2 (Bayer Laboratories, Elkhart, IN, USA). Hyperglycemia was defined as plasma glucose greater than 16.7 mmol/L. Subsequent tests for hyperglycemia were performed at 2, 6, 10 and 18 weeks with blood glucose and Glycosylated hemoglobin measurements. Glycosylated hemoglobin (GHb) was determined by affinity chromatography using Gly-Affin GHb columns (Isolab, Inc., Akron, OH, USA). Plasma was also collected at the same time for the measurement of free and total carnitine by a modification of a radio-enzymatic method [10] and non-esterified free fatty acids (FFA) were measured by the method of Duncombe and Rising [11]. Ultra Lente insulin (Eli Lilly Co., Indianapolis, IN, USA) was injected subcutaneously three times a week at a dose (3–5 units/kg) designed to promote weight gain but inadequate to produce normal blood glucose levels. After 2 weeks of hyperglycemia, sixteen of the forty-eight diabetic animals had L-carnitine (1 mg/ml) (Sigma Tau, Rome, Italy) added to their drinking water for the duration of the study. Echocardiographic measurements were recorded as previously described [3] at base line, 2, 6, 10, and 18 weeks. Twenty-four normal animals the same age served as comparative controls. One of the diabetic + carnitine animals died during the first 2 weeks of the protocol. Twelve of the 32 diabetic animals not receiving carnitine died between the 10 and 18 week measurements. Six animals from each group (control, diabetic, diabetic + carnitine) at 25 weeks had echocardiographic measurements before and 5 minutes after the intravenous administration of dobutamine (10 μg/kg/min). All results are expressed as mean ± SEM. Group means were compared, because of multiple measures, by Bonferroni procedure [12]. Pearson correlation coefficients were used to evaluate the relationship of metabolic parameters to the heart rates of these animals.

## Results

**Figure 1:**
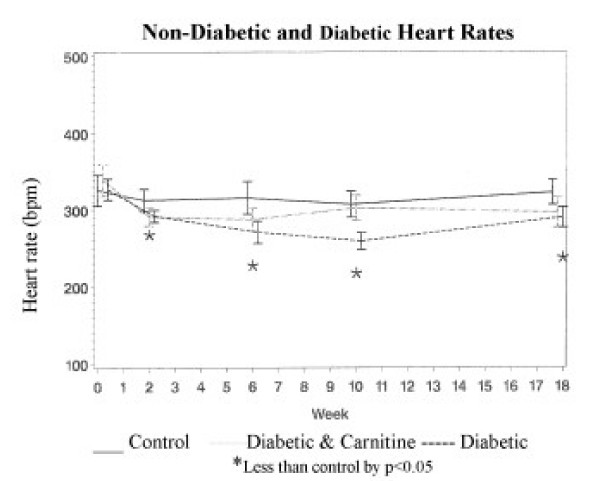
Heart rate in beats/minute [BPM] [mean ± SD] at baseline, 2,6,10 and 18 weeks. * indicates values different from normal control values p < 0.05.

Resting heart rates were reduced in diabetic rats (292 ± 10 bpm) when compared to controls (314 ± 9 bpm) (p < 0.05) after 2 weeks of diabetes. After 18 weeks of diabetes the heart rates (290 ± 19 bpm) remained less than found in the non diabetic control group (324 ± 20 bpm). The free carnitine levels of the diabetic rats (19.1 ± 1.3) were less than those found in control animals (43.7 ± 1.4) (p < 0.01). Carnitine supplementation of the diabetic rats for 16 weeks resulted in normal serum carnitine levels (54.8 ± 4.4 nmol/ml) and heart rates (297 ± 22 bpm). The heart rate decline was first noted 2 weeks after diabetes was induced with STZ. The difference remained significant during the 18 weeks of follow-up in the STZ-D animals. However, the greatest difference in heart rates was noted after 10 weeks of diabetes (259 ± 16 bpm) compared to controls (307 ± 19 bpm). Twelve of the diabetic animals died during the next 8 weeks and the heart rates of the surviving 20 (290 ± 17 bpm) remained less than the non diabetic animals (324 ± 20 bpm), but not different from the diabetic animals treated with supplemental carnitine (297 ± 22 bpm). The 12 animals that died between 10 and 18 weeks had much lower heart rates (222 ± 19 vs. 281 ± 16) (p < 0.008) and serum free carnitine (9.6 ± 3.3 vs. 24.2 ± 3.4) (p < 0.008) at 10 weeks than the STZ-D rats surviving 18 weeks. The STZ-D animals receiving carnitine in their drinking water had mean heart rates begin to increase after week 2 and returned to the non-diabetic control levels by week 10 (figure 1). Dobutamine stress testing in 6 animals from each group resulted in increased heart rates. The heart rate increase with the administration of dobutamine in the diabetic rats (141 ± 8 bpm) was greater than controls (79 ± 9 bpm) (p < 0.05), but the heart rate increase of diabetic rats receiving supplemental carnitine (94 ± 9 bpm) did not differ from control animals.

**Figure 2:**
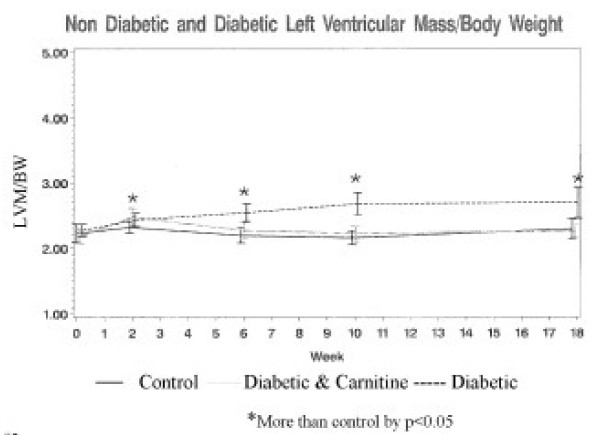
LVM/BW is the ratio of left ventricular mass/total body mass [gm/kg] measured at baseline 2,6,10 and 18 weeks. The values are mean ± SD. * indicates values that differ from control values p < 0.05.

The left ventricular mass/body weight ratio LVM/BW of the diabetic animals (2.7 ± 0.5 gm/kg) was greater than control animals (2.3 ± 0.4 gm/kg) (p < 0.05) after 18 weeks of diabetes. The weight of animals after 18 weeks of diabetes was less than the control animals. The diabetic animals receiving oral carnitine weighed the same as the diabetic animals that did not receive carnitine (table 1) but the LVM/BW (2.3 ± 0.2 gm/kg) was the same as the control animals. The increase of left ventricular mass in relation to body mass of the diabetic animals not receiving carnitine was greater during the 18 weeks of observation, but the weight gain of the diabetic animals was less than the control animals. The increase of left ventricular mass in the diabetic animals receiving oral carnitine was not as great as the un-supplemented diabetic animals and was more consistent with the increase in body mass for those animals. The LVM/BW was greater than control values only for the diabetic animals with low serum carnitine levels (figure 2). The control animals had a mean HbA1c during the 18-week experiment of 5.9% ± 0.8 and the diabetic animals mean HbA1c was 10.9% ± 1.9 (p < 0.0001). The mean HbA1c of the diabetic animals treated with oral carnitine (10.6% ± 2.5), was similar to the diabetic animals not receiving carnitine. The heart rates of all of the rats in this study not receiving supplemental carnitine were inversely correlated to the HbA1c (r = -0.45, p = 0.0001) and directly correlated to the serum free carnitine levels (r = 0.36, p <0.0001). The serum free carnitine levels of the control and the diabetic animals without carnitine supplementation were inversely related to HbA1c (r = -0.33, p < 0.04); serum free carnitine in all of the study animals correlated inversely to the free fatty acid (FFA) levels (r = -0.37, p < 0.02). The heart rate association to HbA1c and serum free carnitine was not found in the normal control or diabetic animals receiving carnitine supplementation. The heart rates of the STZ-D animals, however, correlated directly with the free carnitine levels (r = 0.41, p < 0.0001), and inversely to HbA1c (r = -0.34, p = 0.0002) with Stepwise regression indicating that free carnitine had the greatest influence upon the heart rate.

**Table 1:** Parameters after 18 weeks of STZ diabetes

Variables	Control [24]	Diabetic [20]	Diabetic +Carnitine [15]
Weight [grams]	802 ± 74	711 ± 39*	715 ± 64*
LVM/BW [Gm/kg body weight]	2.3 ± 0.4	2.7 ± 0.5*	2.3 ± 0.2
Heart rate [beats/minute]	324 ± 20	290 ± 19*	297 ± 22
HbA1c [%]	5.9 ± 0.8	10.9 ± 1.8*	10.6 ± 2.4*
Total Carnitine [nmol/ml]	54.6 ± 6.1	59.3 ± 9.7	70.2 ± 11.3
Free Carnitine [nmol/ml]	43.7 ± 1.4	19.1 ± 1.3*	54.8 ± 4.4
FFA [meq./l]	0.26 ± 0.11	0.54 ± 0.33*	0.41 ± 0.19*

*Different from control p < 0.01

## Discussion

Cardiac function in rats is altered in association with hyperglycemia [3-5]. The resting heart rates of STZ-D rats have been noted to be reduced. This finding has been unexplained, but the generally accepted mechanism is diabetic neuropathy [13-15]. Hyperglycemia is believed to cause peripheral nerve dysfunction [6]. The animals reported in this study had diabetes induced at 26 weeks of life and had an immediate reduction of their resting heart rates after 2 weeks of hyperglycemia. We have previously shown in Wistar rats that hyperglycemia onset at 26 weeks of age does not induce altered peripheral nerve function for the next 6 months [7]. Moreover, those with altered peripheral nerve function at 26 weeks do not improved in response to carnitine supplementation after 26 weeks [16]. The decline in heart rate of the diabetic animals progressed at measures 6 and 10 weeks after streptozotocin induced diabetes (figure 1). Between the 10 week and 18 week measurements, 12 of the 32 animals in this group died, and the mean heart rate for the remaining members of this group increased but continued to be less than the control animals at 18 weeks. It is interesting to note that the diabetic animals that died between 10 and 18 weeks were those with the lowest free carnitine levels and heart rates at the 10 week measurement. This finding suggests that severely reduced carnitine levels may have contributed to the demise of those animals. Although the surviving diabetic animals without carnitine supplementation at 18 weeks continued to have heart rates less than the normal control animals, their heart rates and carnitine levels were closer to normal (figure 1).

The myocardium utilizes both glucose and fatty acids for energy metabolism. An abnormality in myocardial energy metabolism [17] is a reported factor in the development of diabetes-induced heart dysfunction. It has been noted that the myocardium of STZ-D rats has reduced glucose transport [18], and oxidation [19], as well as reduced fatty acid oxidation [20] and reduced ATP production [21]. Under usual circumstances, fatty acid oxidation accounts for 60% – 70%, glucose 40% – 30% of myocardial energy supply. Under aerobic conditions fatty acids are the preferred energy substrate for the heart [22]. The transport of long chain fatty acids across the mitochondrial membrane to the site of beta-oxidation is dependent on carnitine [23]. The importance of carnitine has been demonstrated in clinical studies showing myocardial carnitine deficiency to be associated with biventricular hypertrophy [24] and cardiomyopathy [25]. Carnitine deficiency has been reported in subjects with type 1 diabetes [28]. The likely mechanism is excessive urinary loss in subjects with high blood glucose levels and excessive urine volume, which contains large quantities of organic acids as carnitine esters. Esterification of carnitine reduces the normal renal tubular re-absorption of free carnitine, which increases the daily loss. Reduced levels of carnitine limit the availability of fatty acids in the mitochondria to generate ATP. It has been shown that proprionyl-carnitine increases mitochondrial metabolism of pyruvate with associated improvement of function in the isolated perfused heart of STZ-D rats [26]. The secondary substrate available to generate ATP for myocardial function is glucose. The availability of this substrate to the myocyte is limited by the fixed dose of insulin administered each day to insulin deficient animals. The evidence that these important substrates had limited intracellular availability was the elevated HbA1c [elevated extracellular glucose] and elevated free fatty acids found in the diabetic animals. When the hyperglycemic animals had higher free carnitine levels and lower levels of free fatty acids, their cardiac function normalized. The carnitine treated and untreated diabetic animal in this experiment had the same degree of hyperglycemia as indicated by their HbA1c levels at the end of the experiment. The dose of exogenous insulin for both groups of animals was the same, but the free carnitine levels were higher in the STZ-D animals with oral carnitine supplementation. These animals also had lower levels of extracellular free fatty acids. Those STZ-D rats receiving oral carnitine had heart rates that did not differ from the non-diabetic control animals. Dobutamine stress in these diabetic animals produced an exaggerated heart rate response only in those animals without carnitine supplementation. This observation suggests that availability of energy substrate for the myocardium plays an important role in heart rate regulation beyond autonomic tone. Isolated peripheral nerve dysfunction appears to be an unlikely explanation for the abnormal heart rates at rest and with stress as noted in these STZ-D rats. Centrally mediated or local SA node effects in the diabetic animals are possible explanations for these observations, but the corrective function of carnitine makes that mechanism unclear.

The left ventricular mass/body weight ratio of the diabetic animals was greater than that found in the control animals. This finding could be interpreted as indicative of left ventricular hypertrophy in these animals. The left ventricles of the diabetic animals, however, increased in size at the same rate as those of the control animals; the apparent difference in LVM/BW was the result of reduced body weight gain in the diabetic animals. Failure to gain weight is a common finding in diabetic animals, which results from insufficient insulin for body mass increase. If LVM is normally linked to increasing body mass, then the diabetic animals showed excessive increase in LVM during the period of observation. This was confirmed by the observation that the diabetic animals receiving oral carnitine supplementation gained body weight at the same reduced rate as the carnitine deficient diabetic rats but their LVM/BW was the same as the normal animals. Thus, insulin limited diabetic animals with abundant free carnitine levels may have utilized increased fatty acid metabolism to promote LVM increase in a normal relationship with the animal body mass.

Oral carnitine supplementation in a group of STZ-D rats with the same degree of hyperglycemia (HbA1c 10.9% vs. 10.6%) raised their serum carnitine level more than 2 fold restoring the carnitine to control values (table 1). These carnitine-replete animals had normal resting heart rates after 8 weeks of supplementation and a normal heart rate response to dobutamine stress after 16 weeks of supplementation. The carnitine-supplemented STZ-D animals also had an increase in left ventricular mass more consistent with the increase in body weight noted in the control animals. This finding suggests that the increase in ventricular mass found in the carnitine deficient diabetic animals was excessive. Free fatty acid levels were greater and the free carnitine levels were lower in the diabetic animals than the control animals. Thus, it appears that a deficiency of free carnitine can manifest as elevated free fatty acids, suggesting a reduction of inter-cellular transport. Elevating free carnitine levels in insulin depleted diabetic animals with a fixed and relatively inadequate availability of glucose as a myocardial fuel apparently corrected the defects in myocardial function by providing more intracellular fatty acids as an energy substrate.

This experimental model appears to be an example of diabetic cardiac dysfunction caused by inadequate intracellular substrate in a milieu of excessive concentrations of extra-cellular glucose and fatty acids. This phenomenon can occur in clinical medicine. Carnitine levels should be measured in diabetic patients to insure they have sufficient levels to provide abundant substrate for cardiac function, particularly during times of physical stress. Further work is necessary to determine the contemporary prevalence of carnitine deficiency in diabetic patients. The therapeutic use of carnitine in this and other forms of cardiomyopathy likewise needs evaluation.

## Abbreviations

Streptozotocin induced Diabetes (STZ-D)

Hemoglobin A1c (HbA1c)

Free Fatty Acids (FFA)

Beats per Minute (bpm)

Left Ventricular Mass/Body Weight (LVM/BW)

## Competing interests

The author(s) declare that they have no competing interests.

## Authors' contributions

JIM conceived and designed the study as well as drafting the manuscript.

DDC performed all of the biostatistics and graphs in this study, MAM performed the animal experiments, DDS participated in the study design and interpretation of the data analysis. All authors read and approved the final manuscript.

## References

[B1] Grundy SM, Benjamin IJ, Burke GL, Chait A, Eckel RH, Howard BV, Mitch W (1999). Diabetes and cardiovascular disease – A statement for healthcare professionals from the American heart association. Circulation.

[B2] The Diabetes Control and Complications Trial Research Group (1993). The effect of intensive treatment of diabetes on the development and progression of long-term complications in insulin-dependent diabetes mellitus. N Eng J Med.

[B3] Malone JI, Schocken DD, Morrison AD, Gilbert-Barness E (1999). Diabetic cardiomyopathy and carnitine deficiency. Jour Diab Comp.

[B4] Hoit BD, Castro C, Bultron G, Knight S, Matlib MA (1999). Noninvasive evaluation of cardiac dysfunction by echocardiography in streptozotocin-induced diabetic rats. J Card Fail.

[B5] Van Buren T, Schiereck P, De Ruiter GJW, Gispen WH, De Wildt DJ (1998). Vagal efferent control of electrical properties of the heart in experimental diabetes. Acta Diabetologia.

[B6] Aring AM, Jones DE, Falko JM (2005). Evaluation and prevention of diabetic Neuropathy. AM FAM Physician.

[B7] Malone JI, Lowitt S, Korthals JK, Salem A, Miranda C (1996). The effect of hyperglycemia on nerve conduction and structure is age dependent. Diabetes.

[B8] Mamoulakis D, Galanakis E, Dionyssopoulou E, Evangeliou A, Sbyrakis S (2004). Carnitine deficiency in children and adolescents with type 1 diabetes. J Diabetes Complications.

[B9] (1985). The guide for the care and use of laboratory animal.

[B10] McGarry JD, Foster DW (1976). An improved and simplified radio-isotopic assay for determination of free and esterified carnitine. J Lipid Res.

[B11] Duncombe WG, Rising TJ (1973). Quantitative extraction and determination of nonesterified fatty acids in plasma. Journal of Lipid Research.

[B12] Bonferroni CE (1936). Teoria statistica delle classi e calcolo delle probabilità. Pubblicazioni del R Istituto Superiore di Scienze Economiche e Commerciali di Firenze.

[B13] Schaan BD, Dall'Ago P, Maeda CY, Ferlin E, Fernandes TG, Schmid H, Irigoyen MC (2004). Relationship between cardiovascular dysfunction and hyperglycemia in streptozotocin-induced diabetes in rats. Braz J Med Biol Res.

[B14] McDowell TS, Chapleau MW, Jajduuczak G, Abbund FM (1994). Baroreflex dysfunction in diabetes mellitus I. Selective impairment of parasympathetic control of heart rate. Am J Physiol.

[B15] Dall'Ago P, Fernandes TG, Machado UF, Bello AA, Irigoyen MC (1997). Baroreflex and chemoreflex dysfunction in streptozotocin-diabetic rats. Braz J Med Biol Res.

[B16] Malone JI, Lowitt S, Salem AF (1991). Sorbinil reverses peripheral diabetic neuropathy after 4 or 8, but not 16 weeks of untreated experimental Diabetes. Clin Res.

[B17] Neely MR, Morgan HE (1974). Relationship between carbohydrate and lipid metabolism and the energy balance of heart muscle. Annu Rev Physiol.

[B18] Garvey WT, Hardin D, Juhaszova M, Dominguez JH (1993). Effects of diabetes on myocardial glucose transport system in rats: implications for diabetic cardiomyopathy. Am J Physiol.

[B19] Chatham JC, Forder JR (1993). A 13C NMR study of glucose oxidation in the intact functioning rat heart following diabetes-induced cardiomyopathy. J Mol Cell Cardiol.

[B20] Oshima M, Higashi S, Kikuchi Y, Fukumitsu M, Ban S, Shiai T, Furui S, Yasukoc (1996). Auutoradiographic study of myocardial fatty acid metabolism in diabetic mouse using 1251-BMIPP. Mippon Igaku Hoskasen Gakkai Zasshi.

[B21] Stroedter D, Schmidt T, Bretzel RG, Federlin K (1995). Glucose metabolism and left ventricular dysfunction are normalized by insulin and islet transplantation in mild diabetes in the rat. Acta Diabetol.

[B22] Ponsot E, Zoll J, N'Guessan B, Ribera F, Lampert E, Richard R, Veksler V, Ventura-Clapier R, Mettauer B (2005). Mitochondrial tissue specificity of substrates utilization in rat cardiac and skeletal muscles. J Cellular Physiology.

[B23] Bremer J (1983). Carnitine-metabolism and functions. Physiol Rev.

[B24] Waber LJ, Valle D, Neill C, DiMauro S, Shug A (1982). Carnitine deficiency presenting as familial cardiomyopathy: a treatable defect in carnitine transport. J Pediatr.

[B25] Fang ZY, Prins JB, Marwick TH (2004). Diabetic cardiomyopathy: evidence, mechanisms, and therapeutic implications. Endocr Rev.

[B26] Broderick TL, Paulson DJ, Gillis M (2004). Effects of propionyl-carnitine on mitochondrial respiration and post-ischaemic cardiac function in the ischaemic underperfused diabetic rat heart. Drugs RD.

